# Exploring relationships between whole carcass condemnation abattoir data, non-disease factors and disease outbreaks in swine herds in Ontario (2001–2007)

**DOI:** 10.1186/1756-0500-7-185

**Published:** 2014-03-28

**Authors:** Andrea L Thomas-Bachli, David L Pearl, Robert M Friendship, Olaf Berke

**Affiliations:** 1 Department of Population Medicine, Ontario Veterinary College, University of Guelph, Guelph, Ontario, Canada; 2 Department of Mathematics and Statistics, University of Guelph, Guelph, Ontario, Canada

## Abstract

**Background:**

Improving upon traditional animal disease surveillance systems may allow more rapid detection of disease outbreaks in animal populations. In Ontario, between the years 2001 – 2007, widespread outbreaks of several diseases caused major impacts to the swine industry. This study was undertaken to investigate whether whole carcass condemnation data of market pigs from provincial abattoirs from 2001 – 2007 could have provided useful information for disease surveillance of Ontario swine. The objective was to examine the suitability of these data for detection of disease outbreaks using multi-level models and spatial scan statistics. We investigated the ability of these data to provide spatially-relevant surveillance information by determining the approximate distance pigs are shipped from farm to provincial abattoirs in the province, and explored potentially biasing non-disease factors within these data.

**Results:**

Provincially-inspected abattoirs in Ontario were found to be located in close proximity to the hog farms of origin. The fall season and increasing abattoir capacity were associated with a decrease in condemnation rates. Condemnation rates varied across agricultural regions by year, and some regions showed yearly trends consistent with the timing of emergence of new disease strains that affected the Ontario swine population. Scan statistics identified stable clusters of condemnations in space that may have represented stable underlying factors influencing condemnations. The temporal scans detected the most likely cluster of high condemnations during the timeframe in which widespread disease events were documented. One space-time cluster took place during the beginning of the historical disease outbreaks and may have provided an early warning signal within a syndromic surveillance system.

**Conclusions:**

Spatial disease surveillance methods may be applicable to whole carcass condemnation data collected at provincially-inspected abattoirs in Ontario for disease detection on a local scale. These data could provide useful information within a syndromic disease surveillance system for protecting swine herd health within the province. However, non-disease factors including region, season and abattoir size need to be considered when applying quantitative methods to abattoir data for disease surveillance.

## Background

There is increasing global interest in improving animal disease surveillance to ensure timely detection of emerging disease outbreaks [1]. Syndromic surveillance is defined as surveillance using health-related data that precedes a diagnosis and signals a sufficient probability of an outbreak, to warrant further public or animal health response [2]. The use of pre-diagnostic data sources might detect naturally and non-naturally-occurring outbreaks sooner than would be possible with traditional diagnostic data [3]. Syndromic surveillance is recommended as an adjunct to traditional disease monitoring and surveillance programs. Although syndromic surveillance systems were originally developed to detect human disease outbreaks, these systems are increasingly being considered for their potential as early warning signals of disease outbreaks in livestock populations, for animal health and public health purposes [4-8].

Inspection of animals pre- and post-slaughter in abattoirs functions to ensure the safety of meat products for human consumption [9], and has also been used for active surveillance of animal and zoonotic diseases [10-12]. There is evidence supporting the sensitivity of meat inspection for detecting specific diseases in animals [13,14], and condemnation rate information from abattoirs has been used to guide control strategies for common infectious diseases in pigs [15]. Provincially-inspected abattoirs in Ontario have the potential to provide information into a syndromic surveillance system for improved detection of disease outbreaks in Ontario finisher herds. However, the accuracy and validity of this type of data for detection of animal disease outbreaks has not been well-documented.

Swine herds in Ontario were affected by several important health events during recent years. Most notable was the emergence in the autumn, 2004 of a strain of porcine circovirus type II (PCV-2), restricted fragment length polymorphism (RFLP) pattern 321, which coincided with an outbreak of a severe form of Porcine Circovirus-Associated Disease (PCVAD). Up to 50% mortality in some grower-finisher pig herds was documented [16] and losses continued until the spring of 2006 when a new vaccine to protect against PCV-2 was implemented on Ontario pig farms [17]. Around the same time period, an outbreak of a more severe form of Porcine Reproductive and Respiratory Syndrome virus (PRRS) occurred, which continued until late 2006 [18], and in the spring of 2005, a triple reassortant subtype H3N2 of swine influenza type A virus (SIV) swept through Ontario pig herds by the summer of 2005 [19]. As a result of these major disease events, there has been attention directed at enhancing surveillance of swine herd health in this province.

The objective of our study was to explore the suitability of whole carcass condemnation data from provincially-inspected abattoirs in Ontario for incorporation into a syndromic disease surveillance system for early detection of new, emerging or re-emerging diseases in finisher hog herds. We examined whether the general proximity of swine operations to provincially-inspected abattoirs would support spatial analysis of abattoir data for disease surveillance. To better understand sources of bias or variables that would require consideration in condemnation data analysis for disease outbreak detection, non-disease factors that influence whole hog condemnations were investigated. In addition, we wanted to assess the performance of the scan statistic on unadjusted data to determine whether clusters of high whole carcass condemnations would be detected that were consistent with known large-scale historical health events.

## Methods

### Data sources and database management

**Figure 1:**
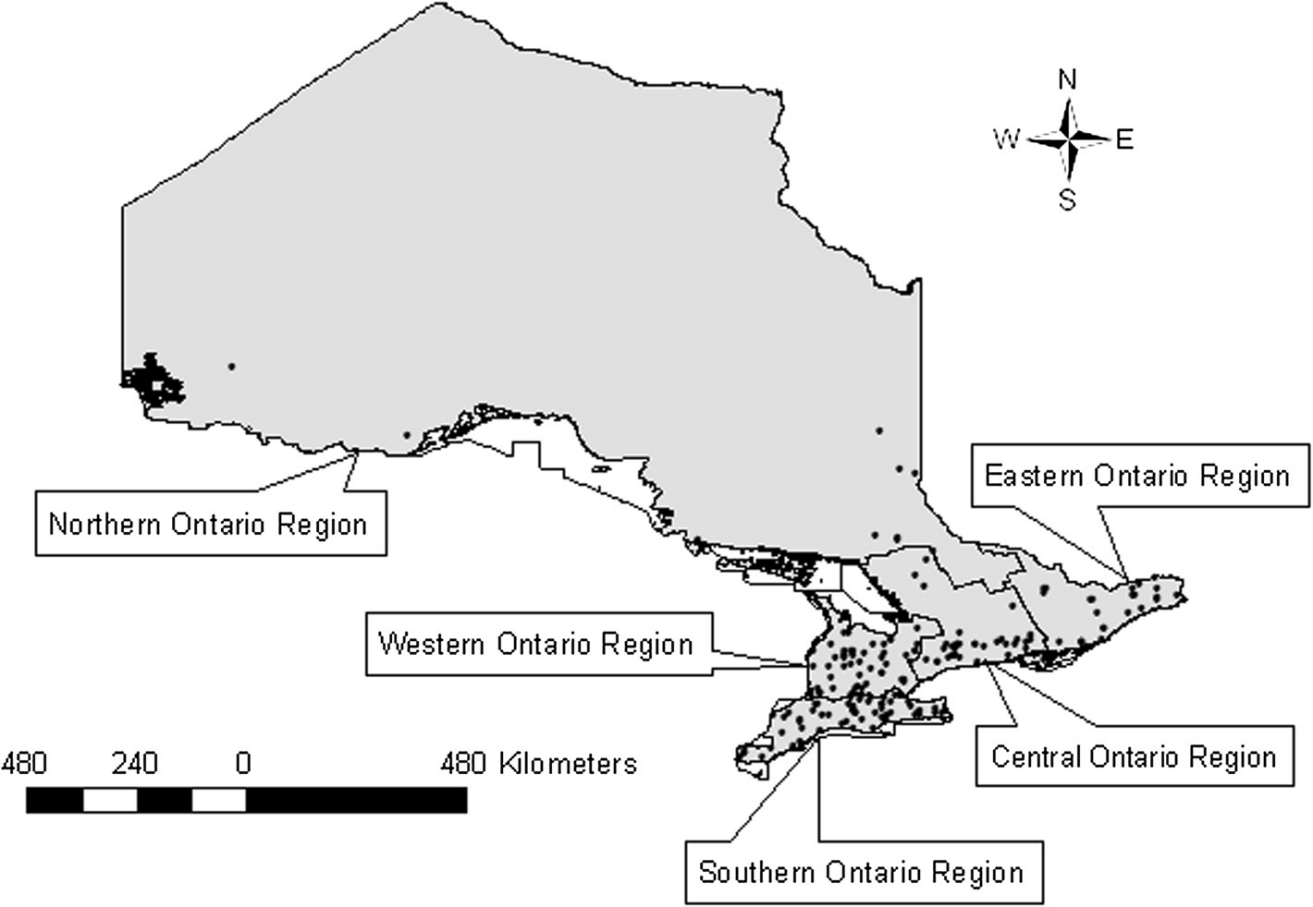
Ontario Provincial abattoirs which processed market hogs between 2001 – 2007 and census agricultural region.

We obtained data on the daily numbers of market hogs slaughtered and whole carcasses condemned by veterinary inspectors between 2001 – 2007 in all provincially-inspected abattoirs, and their geographic locations across Ontario with permission from the Ontario Ministry of Agriculture, Food and Rural Affairs (OMAFRA). Boundary files regarding the so-called census agricultural regions in Ontario (CAR) were obtained from Statistics Canada [20]. The locations of abattoirs were digitally layered over a map of Ontario (Figure 1), and linked to corresponding agricultural regions. Agricultural regions for the province of Ontario are classified as: Central, Eastern, Northern, Southern, and Western Ontario. Provincial weekly hog stock prices over the study period were obtained from Agriculture Canada [21]. Quarterly median hog stock price was derived from published weekly hog stock prices [21]. Seasons were defined as follows: winter (Jan - Mar), spring (Apr – June), summer (July – Sept), and autumn (Oct – Dec). The total number of hogs processed per year and the number of weeks an abattoir processed at least one hog were calculated. Data were merged into a common dataset using Stata 10.0 (StataCorp, College Station, TX, USA).

### Shipping distance estimation

**Figure 2:**
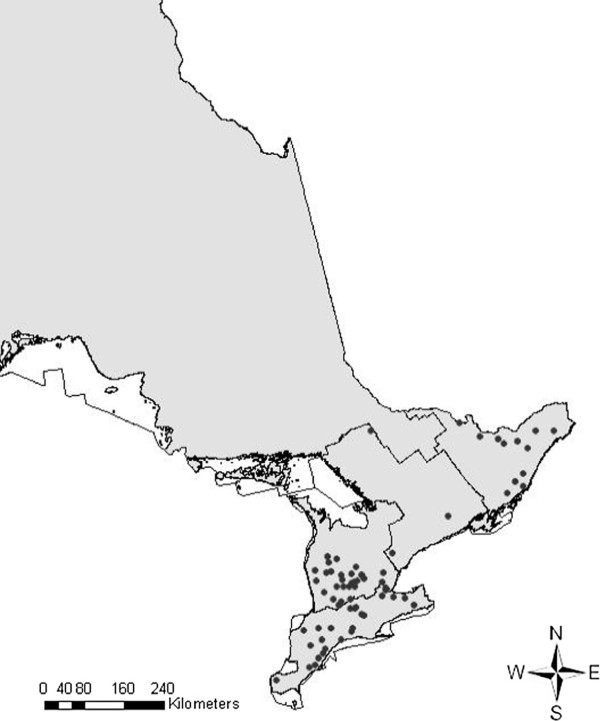
Map representing the distribution of abattoirs where average shipping distance was estimated.

A subset of the data, for which a sample was submitted for laboratory testing and included information about the location of the farm of origin, was used to estimate the typical distance pigs were shipped to slaughter and the range in these distances using the Haversine distance formula [22]. Geocoding software GeoPinpoint Canada (DMTI Spatial Inc., Markham, Ontario, Canada) was used to obtain a latitude and longitude for the postal codes provided for each farm of origin. We plotted the abattoirs where this data was collected into the ArcGIS map (Figure 2) to ensure that good coverage over Ontario was included in the sample.

### Statistical models

Due to issues concerning the lack of independence between repeated measurements from abattoirs, a Poisson model and a negative binomial model, each with a random intercept for abattoir, were constructed. All statistical analyses were performed using STATA Intercooled 10.0 (StataCorp, College Station, Texas). The dataset was collapsed to a quarterly level for modeling. The effects of the following variables on quarterly condemnation rate were studied: year, season, census agricultural region, median market hog stock price, number of weeks each abattoir processed swine carcasses per year, and total number of hog carcasses processed per year. To assess the linearity of the relationship between the natural log transformed condemnation rate and the continuous variables (median stock price, total number of hogs processed per year, and the number of weeks an abattoir processed hogs per year), lowess (locally weighted) curves were examined [23]. Any continuous variable found to have a non-linear relationship with the log of condemnation rate was categorized if it could not be linearized with an appropriate transformation or modeled as a curvilinear relationship with the addition of a quadratic term to the model. In order to avoid collinearity, we examined a Spearman’s rank correlation coefficient (r_s_) between each pair of variables, and if any two variables were found to be strongly correlated (ie., r_s_ ≥ 0.8) the variable considered most informative was used in subsequent models [23].

Multivariable models were built using a liberal significance level of alpha < 0.20 in the univariable statistical analyses. All significant variables from the univariable statistical analyses were included, and a manual backward elimination procedure was used to create a final main effects model. Likelihood ratio tests were used to evaluate the significance of each variable. Following the backward elimination of non-significant main effects, likelihood ratio tests were used to compare nested models with and without the following interaction terms: year and census agricultural region, year and season, and census agricultural region and season. A variable was considered to be a confounder and was kept in the model if it was not an intervening variable and its removal resulted in a 20% or greater change in any of the coefficients of statistically significant variables [23]. Coefficients were exponentiated to report as incident rate ratios (IRRs) for each unit change in the predictor variables. All tests in the final model were two-tailed and variables were considered statistically significant at a level of alpha ≤ 0.05. Contrasts and predicted outcomes for specific covariate patterns were evaluated graphically after calculating point estimates for linear combinations of predictors using the lincom command in Stata.

The Poisson model was built using adaptive Gaussian quadrature [24] with the multi-level mixed effects model for count data using the xtmepoisson command in Stata. The negative binomial model was built with the random effects overdispersion model for count data using the xtnbreg command in Stata. The overdispersion present in the data is accommodated by this model by allowing the overdispersion parameter to vary randomly following a beta distribution [24,25]. For each model, the offset was the log number of hogs processed at the abattoirs per quarter and the outcome was the number of whole carcasses condemned per quarter. Akaike’s Information Criterion (AIC) measure was used to assess which of these models provided the best fit [24]. Depending on the best-fitting model and options available for calculating residuals, the following were to be assessed: crude, Pearson, Anscombe, and deviance residuals. Pearson and deviance residuals were examined for large values that would indicate outliers and all residuals were plotted against the predicted outcomes for evaluation of model fit.

### Retrospective scan statistics

Spatial, temporal, and space-time scan tests were performed to detect clusters of high condemnations during the entire study period (2001 – 2007) using SaTScan version 8.0 software [25]. Spatial scan tests were also applied for each year using data containing numbers of hogs condemned and processed daily at the abattoirs. SaTScan software performs scan statistics by creating scanning windows that vary continuously in space and/or time and comparing the number of observed to expected cases inside each window [25]. A relative risk (RR) and log likelihood ratio for spatial and temporal scan tests and an observed/expected (O/E) ratio for the space-time scan tests were estimated based on Poisson, Bernouilli, and space-time permutation models, respectively [25]. The Bernoulli model was used rather than the Poisson model for the temporal scan tests as a method to avoid potential biases that might be introduced during time periods where abattoirs did not process hogs due to closures on holidays and weekends. The space-time permutation model compares cases within the window to the number that would be expected if there were no space-time interaction, while automatically adjusting for purely spatial or temporal clusters [25]. The most likely cluster is determined as the window with the maximum likelihood based on a likelihood ratio test statistic, and Monte Carlo replications are performed to determine the significance level [26]. For the spatial and spatio-temporal models, any secondary clusters and their level of significance are also reported [25].

Case and population datasets are used for the Poisson model-based spatial scan statistic [25], which, for this study, were the daily number of carcasses condemned and the total number processed at each facility, respectively. The Bernoulli-based temporal scans used the same case data and a control dataset [25] comprised of the daily numbers of hogs deemed suitable for human consumption by inspectors. The space-time scan tests require only case data containing the abattoir identification for each condemned animal and the date of condemnation [25]. Geographic coordinates were in latitude and longitude. The maximum scanning window was specified as 50% of the population of animals processed, or population at risk (PAR) for the spatial scans, 50% of the study period for temporal scans, and both 50% of the condemnations and study period for the space-time scan tests. For each temporal and space-time scan test we specified a scanning time aggregation length of 1 day. For significance testing a simulation-based p-value based on 9999 Monte Carlo samples was used for the spatial and temporal scan tests. Due to excessive computational time the number of Monte Carlo samples was reduced to 999 in the space-time situation. We reported clusters with no spatial overlap for our spatial scans. For the space-time scans, we selected a reporting option that allows reporting of overlapping clusters, provided there were no pairs of centers in each others clusters. This allowed the capture of geographically overlapping clusters taking place during different time periods. Space-time clusters that were non-overlapping are presented. All scan statistics were performed in SaTScan 8.0 [25].

### Summary statistics for cluster interpretation

Spatial and space-time scan tests that were significant at a significance level of alpha ≤ 0.05 were added as a layer to the map created in our GIS database for geographical visualization and for linkage with the epidemiological data. Summary statistics were estimated for the abattoirs within the statistically significant clusters, to determine the total number of animals slaughtered and condemned, and rates of condemnations within these clusters. In an attempt to validate our findings, qualitative comparisons were made between the results of our scans and statistical models with reports and publications from the Animal Health Laboratory at the University of Guelph during this period [16,18,19,27-31].

## Results

### Descriptive statistics

During 2001 – 2007, there were a total of 2,264,428 market hogs slaughtered in provincially-inspected abattoirs in Ontario. Of these, 13,225 whole animals were condemned as unfit for human consumption. The highest yearly number of market hogs slaughtered was over 400,000 in 2002, with subsequent years showing that progressively fewer hogs were processed (Table 1). Whole carcass condemnation rates increased in 2004, peaked in 2005, and declined in 2006 and 2007 (Table 1). There was a general decreasing trend in the number of abattoirs present during the study (Table 2). The median number of hogs processed in the abattoirs stayed consistent over the years of the study (Table 2) although there was large variability in the numbers processed. The number of weeks an abattoir processed hogs during any year ranged from 1 – 52 except in 2005, which showed a range of 2 – 52 weeks open (Table 2). Over half of all abattoirs were located in the Southern and Western Ontario agricultural regions, followed in order by Eastern Ontario, Central Ontario and Northern Ontario (Figure 1, Table 3). When condemnation rates were calculated for each individual agricultural region, Southern Ontario was found to have a higher crude condemnation rate in comparison with all other regions, followed by Western Ontario, Central Ontario, Eastern Ontario and Northern Ontario, respectively (Table 3).

**Table 1:** The number of market pigs slaughtered, condemned, and the condemnation rate for every 1000 pigs slaughtered in Ontario’s provincial abattoirs (2001–2007)

**Year**	**Total pigs slaughtered**	**Total pigs condemned**	**Condemnation rate per 1000 pigs (CR)**	**95% Confidence interval of CR**
**2001**	391 401	1 635	4.18	4.13 - 4.23
**2002**	407 460	2 163	5.31	5.09 - 5.53
**2003**	368 946	1 576	4.27	4.16 - 4.38
**2004**	317 456	1 927	6.07	5.80 - 6.34
**2005**	276 114	2 462	8.92	8.74 - 9.10
**2006**	261 726	2 135	8.16	7.81 - 8.50
**2007**	241 325	1 327	5.50	5.20 - 5.79
**2001-2007**	2 264 428	13 225	5.84	5.79 - 5.89

**Table 2:** The number of provincially-inspected abattoirs in Ontario that process market hogs (pigs), the median number and range of market hogs processed, and median number of weeks open for processing per year (2001–2007)

**Year**	**Number of abattoirs per year**	**Median number (range) pigs processed per year**	**Interquartile range of number of pigs processed per year**	**Median number (range) of weeks open per year**	**Interquartile range of number of weeks open per year**
**2001**	154	384 (2–56 902)	161 – 969	43 (1–52)	25 – 48
**2002**	150	387 (1–87 198)	144 – 922	43 (1–52)	27 – 48
**2003**	134	434 (1–100 866)	188 – 849	44 (1–52)	27 – 49
**2004**	128	359 (2–109 754)	117 – 765	43 (1–52)	21 – 49
**2005**	127	336 (3–116 309)	126 – 648	43 (2–52)	18 – 49
**2006**	119	304 (1–107 914)	137 – 692	40 (1–52)	23 – 50
**2007**	106	344 (4 – 104 914)	104 – 785	42 (1–52)	24 – 50

**Table 3:** Descriptive statistics pertaining to Ontario provincially-inspected abattoirs that processed market hogs (pigs) between 2001 - 2007

**Agricultural region**	**Total number of abattoirs (%)**	**Total number of pigs processed**	**Total number of pigs condemned**	**Condemnation rate/1000 pigs processed**
**Central Ontario**	25 (12.8)	120 424	429	3.56
**Eastern Ontario**	40 (20.4)	59 798	166	2.78
**Northern Ontario**	11 (5.6)	21 701	42	1.94
**Southern Ontario**	67 (34.2)	1 141 972	8 123	7.11
**Western Ontario**	53 (27.0)	920 531	4 446	4.83
**Total**	196	2 264 426	13 206	Average = 5.83

### Shipping distance estimation

There were 664 samples submitted for laboratory testing from 42 of the 196 abattoirs present during 2001–2007 (Figure 2). From this subset of data, the median distance shipped from farm to abattoir was 36 km, with 75% and 25% of all farms within 83 km and 17 km range from the abattoir, respectively.

### Statistical models

#### Univariable models

The total number of hogs processed per year and the number of weeks an abattoir processed hogs per year were found to be highly correlated based on a Spearman’s correlation coefficient r_s_ = 0.81. We chose to build the model using the variable total hogs processed per year as a surrogate measure of abattoir processing capacity, and modeled this variable on a logarithmic scale to achieve a linear relationship with the outcome. Univariable random-effects negative binomial models revealed the following variables to have significant associations with condemnation rate, based on a likelihood ratio (LR) test: year (*P* <0.01), season (*P* <0.01), census agricultural region (*P* <0.01), Log_10_ total number of hogs processed per year (*P* <0.01), and median quarterly market hog stock price (*P* <0.01) (Table 4). In comparison to 2001, 2004 and 2005 were associated with significantly higher whole carcass condemnation rates (Table 4). The fall season was associated with significantly lower condemnation rates in comparison with winter (Table 4), spring (IRR = 1.19, *P* < 0.01) and summer (IRR = 1.15, *P* < 0.01).

**Table 4:** Univariable random effects negative binomial model

**Variable**	**IRR**	**Standard error**	***P*-value**	**95% confidence interval**
**Year**				
**2001**	(Referent)			
**2002**	1.02	0.05	0.71	0.92 – 1.13
**2003**	1.10	0.06	0.07	0.99 – 1.22
**2004**	1.23	0.07	<0.001	1.11 – 1.36
**2005**	1.30	0.07	<0.001	1.17 – 1.45
**2006**	0.96	0.06	0.49	0.86 – 1.08
**2007**	0.91	0.05	0.11	0.81 – 1.02
**Season**	(Referent)			
**Winter**	1.07	0.04	0.08	0.99 – 1.16
**Spring**	1.04	0.04	0.33	0.96 – 1.12
**Summer**	0.90	0.04	0.01	0.83 – 0.98
**Fall**				
**Census agriculture region**				
**Central Ont.**	(Referent)			
**Eastern Ont.**	1.25	0.28	0.33	0.80 – 1.94
**Northern Ont.**	0.92	0.29	0.78	0.49 – 1.69
**Southern Ont.**	2.37	0.41	<0.001	1.69 – 3.33
**Western Ont.**	0.96	0.18	0.81	0.67 – 1.37
**Log**_**10**_**total processed per year**	0.67	0.02	<0.001	0.63 – 0.70
**Median quarterly** **stock price**	1.003	0.0006	<0.001	1.002 – 1.004

Univariable random intercept negative binomial model with abattoir as a random effect, modeling the association between condemnation rates in provincial abattoirs in Ontario and year, season, census agricultural region, log_10_ total hogs processed per year, and median quarterly hog stock price.

#### Multivariable models

**Figure 3:**
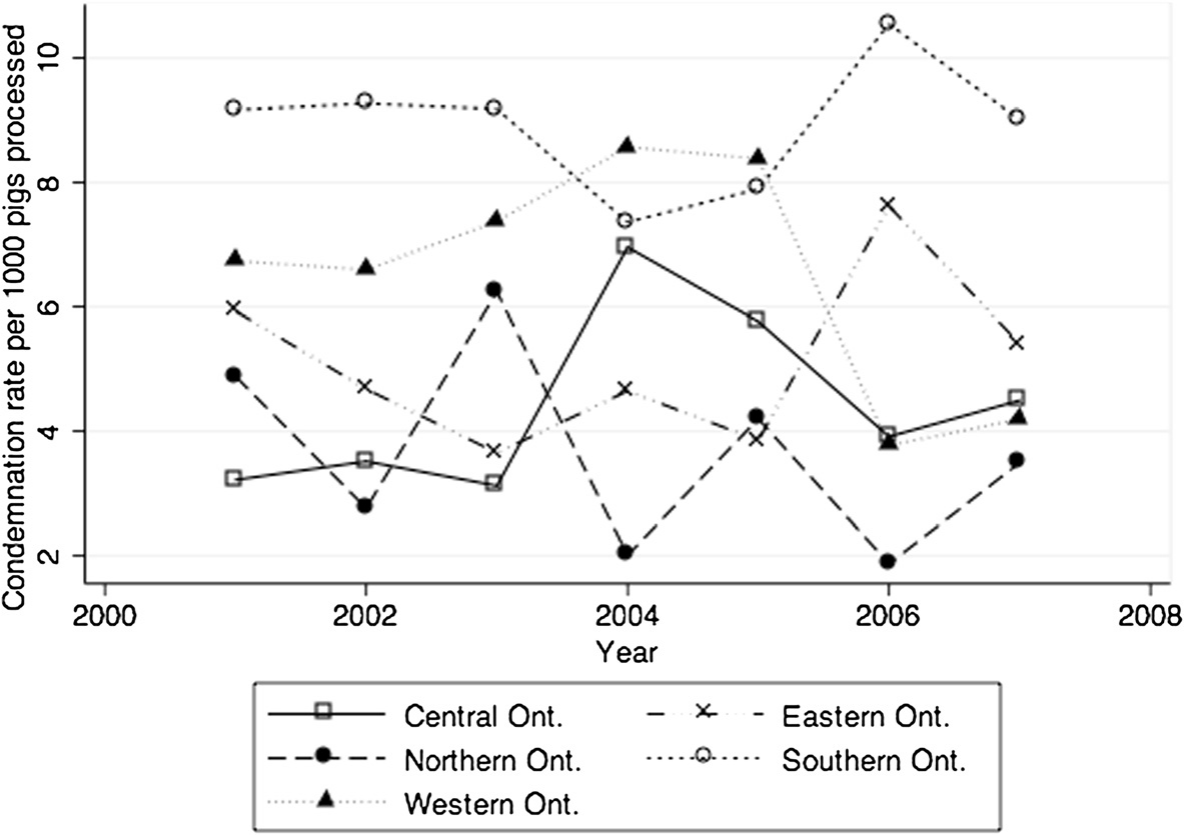
**Graphical representation of the predicted values for the interaction between year and census agricultural region.** Reference season set at winter and total number of pigs processed set at the median provincial value.

A negative binomial model with a random intercept for abattoir was the best fitting multivariable model according to the AIC values for the models (negative binomial model = 8967.9, Poisson model = 9224.8). All subsequent results will pertain to this model (Table 5). The final fitted model showed that, after accounting for all significant variables, condemnation rates were lower for abattoirs with larger processing capacity (Table 5). Fall was associated with lower condemnation rates in comparison with all other seasons (Table 5). Upon examination of the predicted condemnation rates for each combination of census agricultural region and year of study (Figure 3), it was observed that, when all other variables were held constant with the reference season set at winter and total number of pigs processed set at the median provincial value, there were higher rates generally for abattoirs located in the Southern Ontario region, and the rates across each region varied by individual year with peaks occurring in 2004 in Central and Western Ontario, and 2006 in Southern and Eastern Ontario (Figure 3). Plots of the crude residuals against predicted outcomes did not result in identification of large residuals.

**Table 5:** Multivariable random intercept negative binomial model

**Variable**		**IRR**	**P-value**	**Confidence interval**
**Year**	2001	(Referent)		
	2002	1.12	0.66	0.67 – 1.89
	2003	0.99	0.97	0.57 – 1.71
	2004	2.01	0.01	1.22 – 2.39
	2005	1.65	0.06	0.99 – 2.76
	2006	1.13	0.66	0.65 – 1.98
	2007	1.36	0.28	0.78 – 2.35
**Season**	Fall	(Referent)		
	Winter	1.10	0.03	1.01 – 1.20
	Spring	1.19	<0.001	1.10 – 1.29
	Summer	1.17	<0.001	1.08 – 1.27
**CAR**	Central Ontario	(Referent)		
	Eastern Ontario	1.52	0.21	0.79 – 2.89
	Northern Ontario	1.33	0.54	0.55 – 3.26
	Southern Ontario	2.77	<0.001	1.70 – 4.50
	Western Ontario	1.62	0.06	0.98 – 2.70
**Log**_**10**_**total processed per year**		0.82	<0.001	0.76 – 0.88
**Year x CAR**	2001 × Central Ont.	(Referent)		
	2002 × Eastern Ont.	0.70	0.37	0.33 – 1.52
	2002 × Northern Ont.	0.48	0.26	0.13 – 1.73
	2002 × Southern Ont.	0.96	0.89	0.56 – 1.65
	2002 × Western Ont.	0.83	0.50	0.47 – 1.45
	2003 × Eastern Ont.	0.60	0.24	0.25 – 1.41
	2003 × Northern Ont.	1.21	0.73	0.41 – 3.60
	2003 × Southern Ont.	1.09	0.77	0.62 – 1.92
	2003 × Western Ont.	1.05	0.86	0.59 – 1.89
	2004 × Eastern Ont.	0.37	0.02	0.16 – 0.87
	2004 × Northern Ont.	0.20	0.02	0.05 – 0.80
	2004 × Southern Ont.	0.40	<0.001	0.24 – 0.66
	2004 × Western Ont.	0.86	0.58	0.51 – 1.46
	2005 × Eastern Ont.	0.36	0.03	0.14 – 0.92
	2005 × Northern Ont.	0.49	0.22	0.15 – 1.55
	2005 × Southern Ont.	0.56	0.03	0.33 – 0.95
	2005 × Western Ont.	1.10	0.74	0.64 – 1.89
	2006 × Eastern Ont.	1.07	0.88	0.45 – 2.50
	2006 × Northern Ont.	0.30	0.15	0.06 – 1.56
	2006 × Southern Ont.	1.01	0.98	0.56 – 1.80
	2006 × Western Ont.	0.73	0.31	0.40 – 1.33
	2007 × Eastern Ont.	0.63	0.32	0.25 – 1.57
	2007 × Northern Ont.	0.47	0.30	0.11 – 1.98
	2007 × Southern Ont.	0.72	0.27	0.41 – 1.28
	2007 × Western Ont.	0.69	0.22	0.38 – 1.24

Modeling the association between whole carcass condemnation rate (IRR) and year, season, census agricultural region (CAR), total number of market hogs (pigs) processed per year, and the interaction effect between census agricultural region and year.

#### Retrospective scan statistics

**Figure 4:**
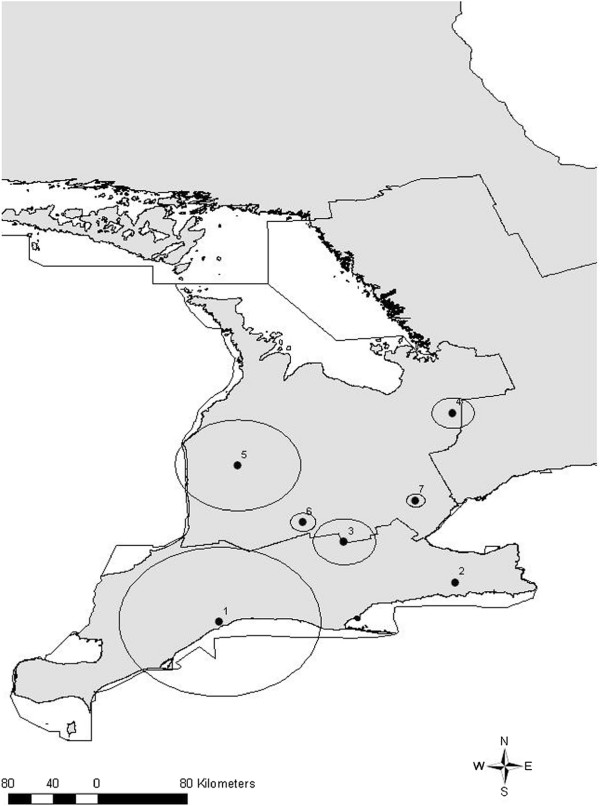
**Map of spatial clusters of high whole carcass condemnations based on a spatial scan of the entire study period (2001 – 2007).** Satscan settings were specified as Poisson model with 50% of the population at risk (total market pigs slaughtered) as the maximum spatial scanning window. Cluster centroids are indicated by solid dots and boundaries are indicated by circles, numbered in descending order from the most likely to least likely.

**Figure 5:**
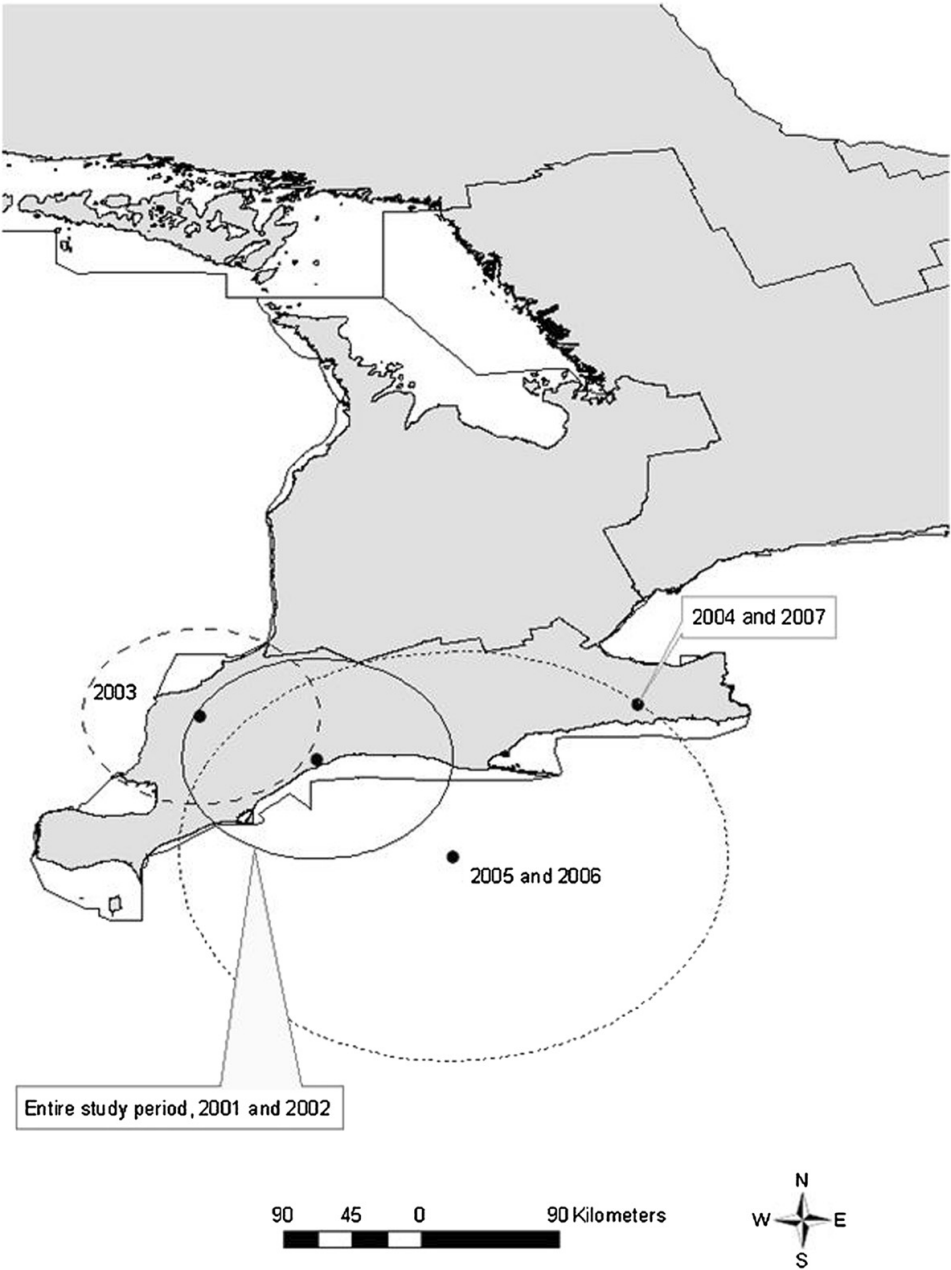
**Map of spatial clusters of high whole carcass condemnations based on a spatial scan of each individual year from 2001 – 2007.** Satscan settings were specified as Poisson model with 50% of the population at risk (total market pigs slaughtered) as the maximum spatial scanning window. Cluster centroids are indicated by solid dots and boundaries are indicated by solid and hashed circles.

Spatial scan tests performed over the entire study period revealed 7 statistically significant (*P* < 0.05) non-overlapping clusters of varying sizes including 2 clusters containing single abattoirs (Figure 4, Table 6). The most likely cluster was located in Southern Ontario (Figure 4, Cluster 1). Two other significant non-overlapping clusters were detected in Southern Ontario, 4 in Western Ontario, and 1 was located in Central Ontario (Figure 4, Clusters 2–7). When spatial scan tests were performed by individual years there were a number of similar and identical clusters to those detected when the entire period was scanned (Table 7, Figure 5).

**Table 6:** Summary of spatial clusters* of high condemnation rates by entire study period (2001 – 2007)

**Cluster**	**Total number of abattoirs**	**Radius (km)**	**Total number of pigs condemned**	**Total number of pigs processed**	**CR/1000 pigs**	**RR**	***P*-value**
**1**	21	66.81	3 446	103 116	33.42	6.85	0.0001
**2**	1	0	1 895	34 109	55.56	10.24	0.0001
**3**	5	20.53	320	12 198	26.23	4.32	0.0001
**4**	3	13.71	130	3 728	34.87	5.69	0.0001
**5**	2	8.21	55	2 790	19.71	3.20	0.0001
**6**	1	0	47	3 128	15.03	2.44	0.0001
**7**	10	31.20	270	31 210	8.65	1.41	0.001

*Detected with a Poisson model using the spatial scan statistic run over the entire study period (2001 – 2007) with a maximum scanning window of 50% of abattoirs and significance level alpha ≤ 0.05.

Descriptive statistics indicating total market hogs (pigs) condemned, processed, corresponding condemnation rates (CR) per 1000 market hogs processed and relative risks (RR) within these clusters.

**Table 7:** Summary of spatial clusters* of high condemnation rates by individual years

**Year**	**Number of abattoirs**	**Radius (km)**	**Number of pigs condemned**	**Number of pigs processed**	**Condemnation rate/1000 pigs**	**Relative risk (RR)**
**2001**	14	66.81	316	11 821	26.73	7.69
**2002**	17	65.03	780	17 543	44.46	12.54
**2003**	11	57.67	207	5 817	35.59	9.44
**2004**	1	0	327	7 031	46.51	9.02
**2005**	25	134.23	1 084	45 187	23.99	4.02
**2006**	21	78.59	1 354	40 081	33.78	9.79
**2007**	1	0	395	6 054	65.25	16.52

*Detected with a Poisson model using the spatial scan statistic run by individual years (2001 – 2007) with a maximum scanning window of 50% of abattoirs and significance level alpha ≤ 0.05.

Descriptive statistics indicating total market hogs condemned, processed, corresponding condemnation rates (CR) per 1000 market hogs processed (pigs) and relative risks (RR) within these clusters.

**Figure 6:**
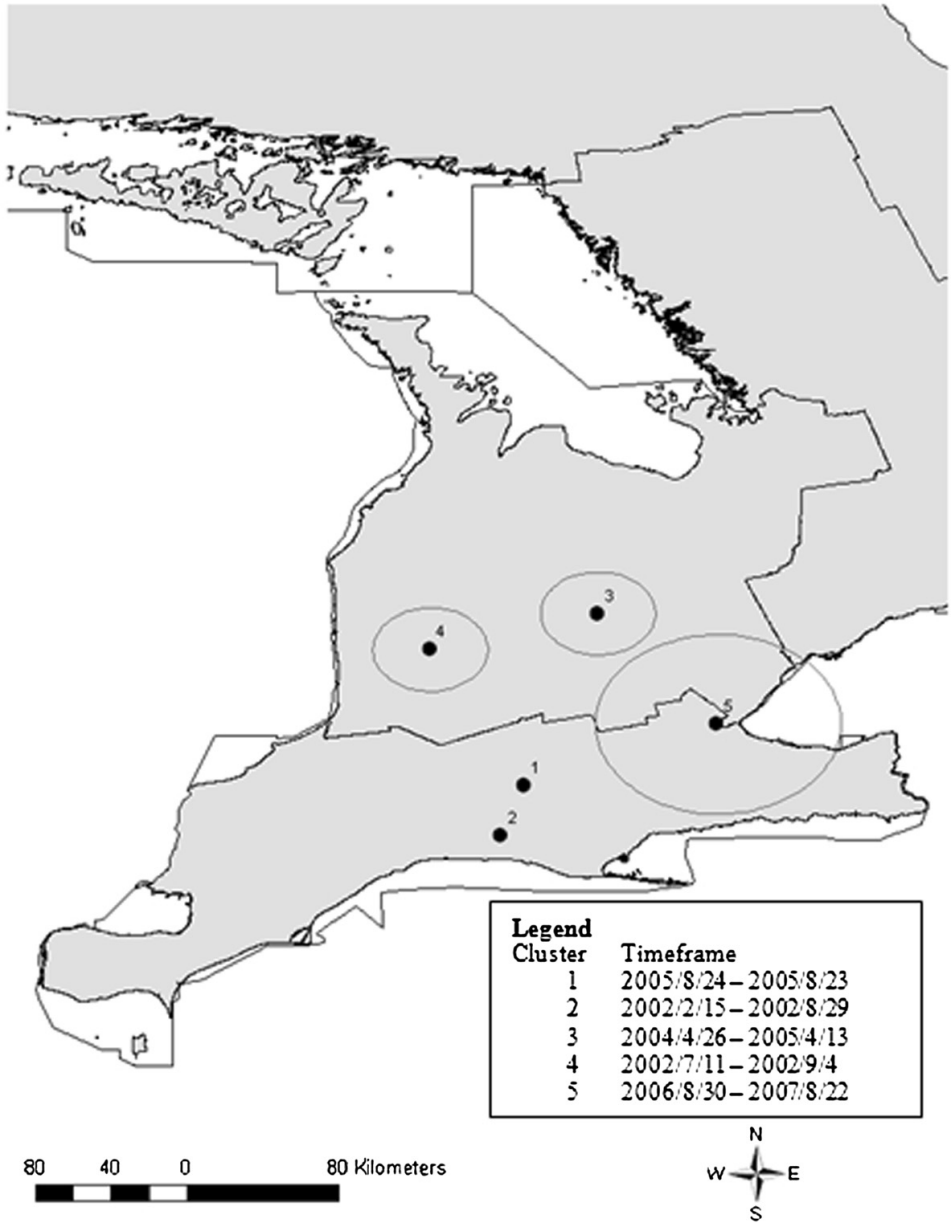
**Map of space-time clusters of high whole carcass condemnations based on a space-time scan of entire study period from 2001 – 2007.** Satscan settings were specified as space-time permutation model with 50% of condemned pigs specified as maximum spatial scanning window and 50% of study period as maximum temporal window, numbered in descending order from most likely to least likely. Cluster centroids are indicated by solid dots and boundaries are indicated by circles, numbered in descending order from the most likely to least likely.

Temporal scan tests of the entire study period identified the most likely temporal cluster (RR = 1.90, *P* ≤ 0.01) occurred from 2004/9/23 - 2006/8/29, with 5,277 whole carcasses condemned among 571,632 slaughtered, resulting in a condemnation rate of 9.23 hogs per 1000 processed. When space-time scan tests were performed for the entire study period there were 5 clusters detected (Figure 6). The first and second most likely clusters were located in Southern Ontario and each contained single abattoirs (Figure 6), with the rest of the clusters located in Southern and Western Ontario. The third most likely cluster containing 3 abattoirs began during the time in which the aforementioned disease outbreaks had started to affect farms in the province [16,18,19]. Space-time clusters were detected in all seasons of the year and ranged from 0 to 47.4 km (Table 8).

**Table 8:** Summary of space-time clusters* of high condemnation rates (2001–2007)

**Cluster**	**Number of abattoirs**	**Date range**	**Radius (km)**	**Number of cases**	**Number processed**	**CR/1000 pigs**	**O/E**	**Y/N**
**1**	1	2005/8/24 - 2006/8/23	0	1 238	30 721	40.3	4.37	Y
**2**	1	2002/2/15 - 2002/8/29	0	494	6 328	78.07	7.63	N
**3**	3	2004/4/16 - 2005/4/13	21.84	1 112	116 112	9.58	2.16	Y
**4**	2	2002/7/11 - 2002/9/4	22.93	83	12 022	6.90	17.88	N
**5**	18	2006/8/30 - 2007/8/22	47.43	450	28 676	15.70	1.83	N

*Clusters were detected using a spatial scan statistic run over the entire study period with the space-time permutation model and significance level alpha ≤ 0.05.

**Y/N refers to positive association with widespread outbreaks of PCVAD, PRRS, and SIV in reference to laboratory reports, whereby scan results within timeframe 2004/08/01 – 2006/12/31 = positive association (Y) and all other results = no association (N).

Descriptive statistics indicating total market hogs condemned, processed, corresponding condemnation rates (CR) per 1000 market hogs processed (pigs), relative risks (RR) within clusters and correspondence with known widespread outbreak timeframe (Y/N)**.

## Discussion and conclusions

The underlying causes for the decline seen in both the number of hogs processed and processing facilities have not been well-documented, however, over the course of the study time period there was also a decline in the number of operating farms within the province of Ontario [32]. These changes may have been a result of low hog prices and economic difficulties experienced by family-operated hog farms over this time period [32]. In contrast to the roughly 200 provincially-inspected abattoirs in Ontario for interprovincial meat trade, there are only 5 federally-inspected abattoirs, which are licensed to process meat for inter-provincial and international trade. Recent research confirms higher condemnation rates in provincial versus federal Ontario abattoirs [6,8]. We also found supportive evidence that provincially-inspected abattoirs are generally located near farms of origin, and these abattoirs may therefore be a source of spatially-representative data for detecting localized outbreaks of disease in Ontario. Although there are guidelines and uniform evaluation criteria for meat inspectors to follow when deciding whether to condemn a carcass or its parts [33], varying whole carcass condemnation rates among abattoirs may reflect individual differences between meat inspectors, environmental conditions, abattoir-specific management practices, geographic differences and other unknown factors. If condemnation data are to be used for syndromic disease surveillance it is important to gain an understanding of the non-disease factors that influence these data.

The results from the negative binomial models revealed several variables that influence whole hog carcass condemnation rates in provincial abattoirs. The different condemnation patterns seen among agricultural regions during the years of the study may have been driven by disease outbreaks, however, regional differences in condemnation rates should also be considered when using these types of data for disease surveillance. Specifically, the higher rate of condemnations for Southern Ontario in comparison with other regions is consistent with trends observed in the crude condemnation rates (Table 3). Regional patterns in condemnations predicted by the model for Western and Central Ontario most closely resemble the trends expected as a result of the known health events that took place during the years of our study. Consideration of geographic differences in condemnation rate could aid interpretation of condemnation clusters and/or be used to set thresholds for detection of temporal or regional clusters within a syndromic surveillance system.

Previous studies have reported seasonal variations in partial condemnations in pigs [34], and condemnations secondary to specific pig diseases [35]. From our multivariable model, whole carcass condemnation rates were lower in the fall, in comparison to all other seasons. This finding could reflect the influence on disease related to the typical five month hog production cycle [36], whereby pigs born during the late spring and early summer seasons reach maturity for marketing in the fall. These pigs may have lower exposure to environmental stressors such as temperature fluctuations and relative humidity during weaning, compared to those weaned in the winter and summer [37,38]. This may make their health more robust against multi-factorial respiratory diseases, which are associated with lower growth rates, reduced productivity, higher morbidity and mortality [38]. Previous findings of increased risk and severity of pneumonic lung lesions at slaughter seen in hogs infected with *Mycoplasma hyopneumoniae* at weaning age [39] demonstrates the influence of early disease on adult health status. Consideration of seasonal effects on condemnation rates is required when these data are interpreted for disease outbreak detection.

The lower condemnation rates associated with larger abattoirs may reflect differences in herd health on the farms of origin that ship to these facilities. This finding agrees with prior research investigating abattoir size and specific organ condemnations in pigs [8]. Another explanation for the association between abattoir size and condemnation rate, may reflect a difference in the quality of inspection related to a reduced length of time available for examination of each hog. A study of broiler flocks in France found higher condemnation rates associated with low slaughter-line processing speeds [40]. Currently, information on slaughter processing speed for the various abattoirs in our study is unavailable to test this hypothesis.

The decision by a producer to ship animals for slaughter may be associated with market value or other trade considerations irrespective of animal health. We did not find an association between market hog stock price and condemnation rates in the multivariable model. However, market hog stock prices may not entirely reflect the profitability to the producer and the decision to ship particular hogs for marketing. The complex relationship between hog stock prices and decisions to ship may be affected by cyclical hog cycles, feed prices and other production costs, currency exchange rates, pork wholesale and retail prices and the producer’s resulting share of profits [41].

We detected clusters of whole carcass condemnations in space, time, and space-time for the entire study period and spatially when the data were analyzed by individual years. Spatial clusters were detected only in Southern, Western and Central Ontario regions when the entire study period was analyzed. When the data was scanned spatiotemporally, clusters were only detected in Southern and Western Ontario. This supports the findings from the descriptive statistics and the multilevel model demonstrating higher condemnation rates in these regions, particularly Southern Ontario. Upon examination of the spatial scan analysis run for individual years, it is apparent that there are some similar spatial clusters that were detected during multiple years, indicating that stable underlying factors may have contributed to these clusters. These factors may reflect abattoir characteristics, including differences between meat inspectors at individual abattoirs, or farm-level factors, including biosecurity, which may influence the health status of pigs in these areas. There were no documented outbreaks in Northern Ontario during 2003 to adequately explain the model-based increase in condemnation rates, however, greater variability in condemnation rates within this region due to fewer hogs processed may explain the variation seen overall within this region across the years.

The temporal cluster of high condemnation rates detected by scan statistics were consistent with the historical emergence of PCVAD, PRRS and swine influenza documented by the AHL. Results of the space-time scan analysis did not detect clusters that could have indicated these outbreaks with the exception of the third most likely cluster. This space-time cluster containing 3 abattoirs, was detected between April 2004 and April 2005, when the aforementioned disease outbreaks were beginning to affect farms in the province [16,18,19]. However, due to the retrospective nature of the study and the lack of source farm information due to privacy constraints, validation of these clusters was not possible. Future prospective studies could evaluate the sensitivity and specificity of cluster detection for disease surveillance of pigs using whole carcass condemnation data by validating clusters with on-farm and laboratory data.

While space-time permutation models have the advantage of controlling for pre-existing purely spatial and temporal clusters, it assumes the background population to be stable in space-time. Using data from all abattoirs across the province, where baseline numbers of animals processed may increase or decrease faster in some areas over others, could have resulted in biased *P*-values, favoring cluster detection. This issue, referred to as population shift bias [25], may be more of a problem when the changes in disease patterns are being examined over longer periods of time, in this study over a number of 5-month production cycles. Employing adjusted space-time scan analysis based on models that account for background population may offer advantages over space-time permutation models on unadjusted data [42].

The most likely temporal cluster of high condemnations was detected during the fall of 2004 until the fall of 2006, which, interestingly, corresponds with the timeframe during which known outbreaks of respiratory illnesses took place in Ontario herds. The introduction of a vaccine to protect against PCV-2 in late 2006 also coincides with the end of the high condemnation rate temporal cluster. These findings are consistent with previous results of temporal scans of PCV-2 positive cases diagnosed at the Animal Health Laboratory, which reported a temporal cluster of high rates among suspected cases tested during 2005 [30], and clusters of low rates of PCV-2 positive cases among those tested during the fall and winter of 2006 for polymerase chain reaction-positive cases [30]. Because it is not clear whether the underlying reasons for the whole carcass condemnations were secondary to lesions or systemic signs of PCVAD, PRRSv and/or swine influenza infection, it is not possible to confirm the results from the spatial, temporal and space-time. The findings of the temporal scan analysis in particular, do appear to correspond with the wide-spread disease event, and suggest that data collected from abattoir inspections may be useful for detecting disease outbreaks earlier than by traditional laboratory diagnostic data.

This preliminary study shows that whole hog carcass condemnation data from provincial abattoirs condemnations may provide useful information for inclusion in a syndromic surveillance system for pigs in the province of Ontario. Interestingly, a study investigating partial condemnations due to lung and kidney pathology did not clearly demonstrate improvement in potential disease detection compared with whole hog carcass condemnation data [16]. In comparison with data pertaining to individual organ condemnations, whole carcass condemnation data may provide a better indicator of overall health status in a population of pigs, making it more suitable for detection of multifactorial disease syndromes. Furthermore, as discussed in our previous paper [16], an important difference between the two data types relates to the requirement for a veterinary inspector to oversee all whole carcass condemnations in provincial abattoirs, whereas a trade inspector is licensed to condemn partial carcasses without direct veterinary oversight [43]. The performance and practicality of incorporating whole carcass condemnation data in a surveillance program warrants further assessment. A prospective study with real-time data that could be validated using laboratory disease incidence data would be beneficial to further investigate the utility of whole carcass condemnation data for disease outbreak detection.

## Competing interests

The authors declare that they have no competing interests.

## Authors' contributions

ALT performed the statistical analysis and drafted the manuscript. DLP, RMF and OB were involved in the conception and design, analysis and interpretation of data, and revising manuscript critically for important intellectual content. All authors read and approved the final manuscript.
